# Multi-analytical and biological insights into *Malcolmia grandiflora* Kuntze

**DOI:** 10.1038/s41598-026-44715-x

**Published:** 2026-04-21

**Authors:** Ahlam Hashem Elwekeel, Enas I. A. Mohamed, Elham Amin, Malak Sadek, Mahytab Mohamed, Marwa H. A. Hassan

**Affiliations:** 1 Department of Pharmacognosy, Faculty of Pharmacy, Beni-Suef University, Beni-Suef, 62514 Egypt; 2 Department of Pharmaceutical Chemistry and Pharmacognosy, College of Pharmacy, Qassim University, Buraidah, 52571 Saudi Arabia; 3 Faculty of Medicine, Beni-Suef University, Beni-Suef, 62514 Egypt

**Keywords:** Anti-inflammatory, Antioxidant, Cytotoxic, *In silico* studies, LC-HRMS, *Malcolmia grandiflora*, Biochemistry, Biotechnology, Cancer, Chemical biology, Chemistry, Drug discovery, Plant sciences

## Abstract

The examination of natural resources represents a promising avenue for discovering bioactive natural products. The genus *Malcolmia* (Brassicaceae) has received minimal phytochemical investigation, despite the recognized pharmacological potential of Brassicaceae members. Herein, *Malcolmia grandiflora* Kuntze was investigated through phytochemical and biological examination of its *n*-hexane and defatted extracts utilizing GC-MS, LC-HRMS, and in vitro assays. The *n*-hexane extract exhibited potent cytotoxicity against colorectal and breast cancer cell lines, whereas the defatted extract displayed selective COX-2 inhibition. Evaluation of the antioxidant efficacy revealed moderate activity of the defatted extract outperforming that of the *n*-hexane extract in the ABTS and FRAP assays, while both extracts displayed weak activity in the DPPH assay. GC-MS analysis revealed 20 metabolites, mainly fatty acid derivatives, esters, and long-chain alcohols. LC-HRMS analysis unveiled diversified metabolic content dominated by flavonoids, sesquiterpenoids, and isothiocyanates. Molecular docking indicated moderate binding affinities of the *n*-hexane metabolites, the disparity noted between the *n*-hexane extract’s cytotoxicity results and its metabolites’ moderate binding affinities suggests the involvement of distinct mechanisms of action. In contrast, docking results underscore flavonoids as promising Cyclin dependent kinases (CDKs) targeted anticancer leads.

**Supplementary Information:**

The online version contains supplementary material available at 10.1038/s41598-026-44715-x.

## Introduction

Brassicaceae (Cruciferae) or mustard family is a large family of dicot plants, constituting around 360 genera and 4000 species. It includes several important crop plants such as cauliflower, cabbage and broccoli known for its valuable contribution in human diet as well as for their health benefits^1^. Additionally, Brassicaceae plants exhibit a diversified content of secondary metabolites, among them glucosinolates, isocyanates, flavonoids, and phenolics are among the most predominant metabolites. These metabolites are noted for their powerful bioactivities e.g. cytotoxic, anti-inflammatory and antimicrobial. Furthermore, their efficacy to quench free radicals, reduce oxidative stress and inhibit inflammatory responses underscored their potential in the treatment of chronic diseases^2–4^. Moreover, Brassicaceae species are rich in dietary fibers, essential vitamins and minerals. These nutrients contribute to overall human health by supporting immune function, bone health, and digestive well-being^5,6^.

The genus *Malcolmia* comprises xerophytic and desert-adapted plants, characterized by diversified metabolic content that confer significant medicinal and nutritional benefits^7^. *M. grandiflora* is a *Malcolmia* species native to Mediterranean and arid regions, it gained interest for its adaptation to harsh environments, which may enhance its production of stress-responsive secondary metabolites analogous to those in well-studied Brassicaceae crops^8^. Within *Malcolmia* genus, *M. grandiflora* has received minimal scientific attention, however other *Malcolmia* species have been previously investigated. *M. africana* was documented for their rich nutritional content of protein, calcium, and potassium content, along with phenolics correlating to its antioxidant capacity^9^. The flavonoids, phenolics and tannins contents of *M. aegyptiaca* and *M. livida*, were quantified and related to their antioxidant properties^7,10^. Moreover, both species displayed analgesic and anti-inflammatory effects in rat models supporting their traditional use for pain relief^7^. Also, the essential oil of *M. cabulica* was reported to exhibit antimicrobial effects against foodborne pathogens and fungi^11^. These reports highlight the genus as a promising source of bioactive metabolites; however, no comprehensive phytochemical profiling or systematic biological evaluation of *M. grandiflora* has yet been conducted, leaving its chemical composition, antioxidant potential, anti-inflammatory effects, and cytotoxic properties largely unexplored. The existing study provides general botanical descriptions, with no integrated analyses combining advanced chemical characterization (e.g., GC-MS, UPLC-MS/MS) with functional assays such as antioxidant, anti-inflammatory, and cytotoxic evaluations.

In vitro testing constitutes a cost effective, high throughput methodology capable of providing a controlled environment for evaluation of anticancer and anti-inflammatory activities of natural extracts. It represents an important step in the drug discovery process, as it provides preliminary data that guide the exploration of prospective bioactive agents from natural sources^12,13^.

In this context, the current research was designed to address the existing knowledge gap *via* conducting a comprehensive phytochemical and biological examination of the lipophilic and defatted extracts of *Malcolmia grandiflora* Kuntze and to correlate the phytochemical content with the observed bioactivities.

## Results and discussion

### TPC and TFC content of *M. grandiflora* Kuntze

The total phenolic content (TPC) of *M. grandiflora* extract was quantified using Folin–Ciocalteu assay. Herein, the result **(**Table 1**)**, expressed as mg gallic acid for 1 g of the dry extract, revealed TPC value as 81.059 ± 5.76 mg GAE/g extract. Previous research on *Malcolmia* species (*M. aegyptiaca* and *M. africana*) has indicated a considerable variation in TPC, this variation might be attributable to ecological factors as well as species-specific genetic makeup. Esmaeili et al., 2014 estimated the TPC of *M. africana* as 225.19 and 208.52 mg GAE/g in aerial parts and seeds of the plant, respectively^14^. On the other side the total flavonoids content (TFC) of the studied plant was evaluated by colorimetric method using aluminum chloride and the current findings estimated the flavonoids content as 17.23 ± 0.454 mg QE/g. Previous research measured the flavonoids content in *M. aegyptiaca* where lower flavonoid content (1.86 mg QE/g) was recorded in this species as compared to the currently studied species *M. grandiflora*^15^.

### GC-MS analysis of the *n*-hexane extract of *M. grandiflora* Kuntze

GC-MS analysis of the *n*-hexane extract **(**Table 2**)** enabled the identification of 20 phytoconstituents, among them: 2-hexadecen-1-ol, 3,7,11,15-tetramethyl-, acetate (21.01%), linolenic acid, methyl ester (17.77%), tetra acetyl-*d*-xylonic nitrile (11.26%), (*Z*)−13-docosenamide (10.33%), and palmitic acid methyl ester (7.07%) were the major detected compounds. Interestingly, 2-hexadecen-1-ol, 3,7,11,15-tetramethyl-, acetate , the major detected compound, was formerly reported for anticancer and antibacterial activities^16^. Linolenic acid is an unsaturated fatty acid essential for human health, previous research indicated that it lowers the risk of coronary artery disease possibly through inhibition of vascular inflammation^17^, moreover, it was found to provide protection against colon cancer^18^. Additionally, tetra acetyl-*d*-xylonic nitrile and (*Z*)−13-docosenamide were reported for anti-tumor and anti-oxidant activity^19^.

### LC-HRMS analysis of *M. grandiflora* Kuntze

**Fig. 1:**
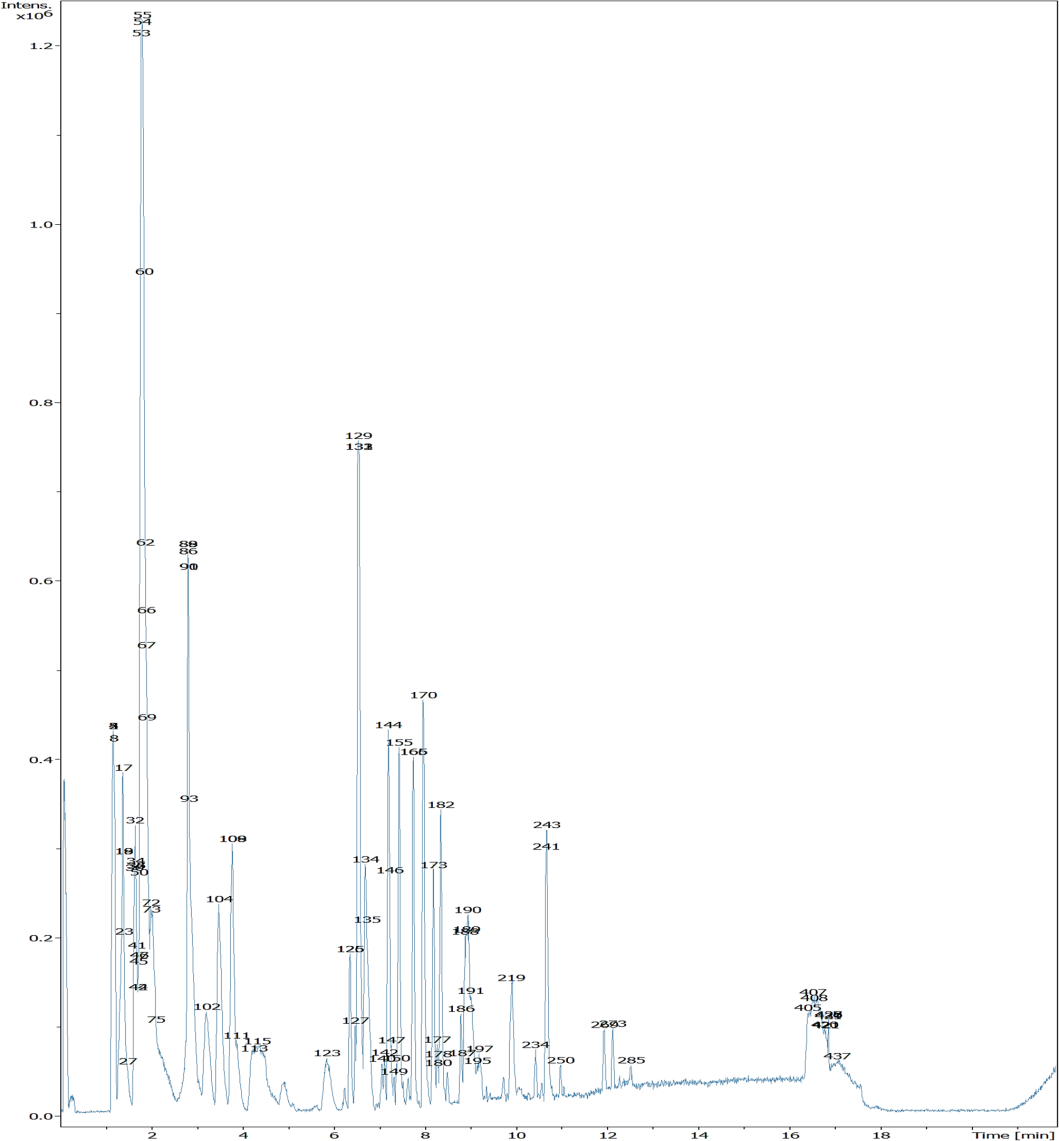
LC-HRMS chromatogram of the dereplicated metabolites of the defatted extract of *Malcolmia grandiflora* Kuntze.

**Fig. 2:**
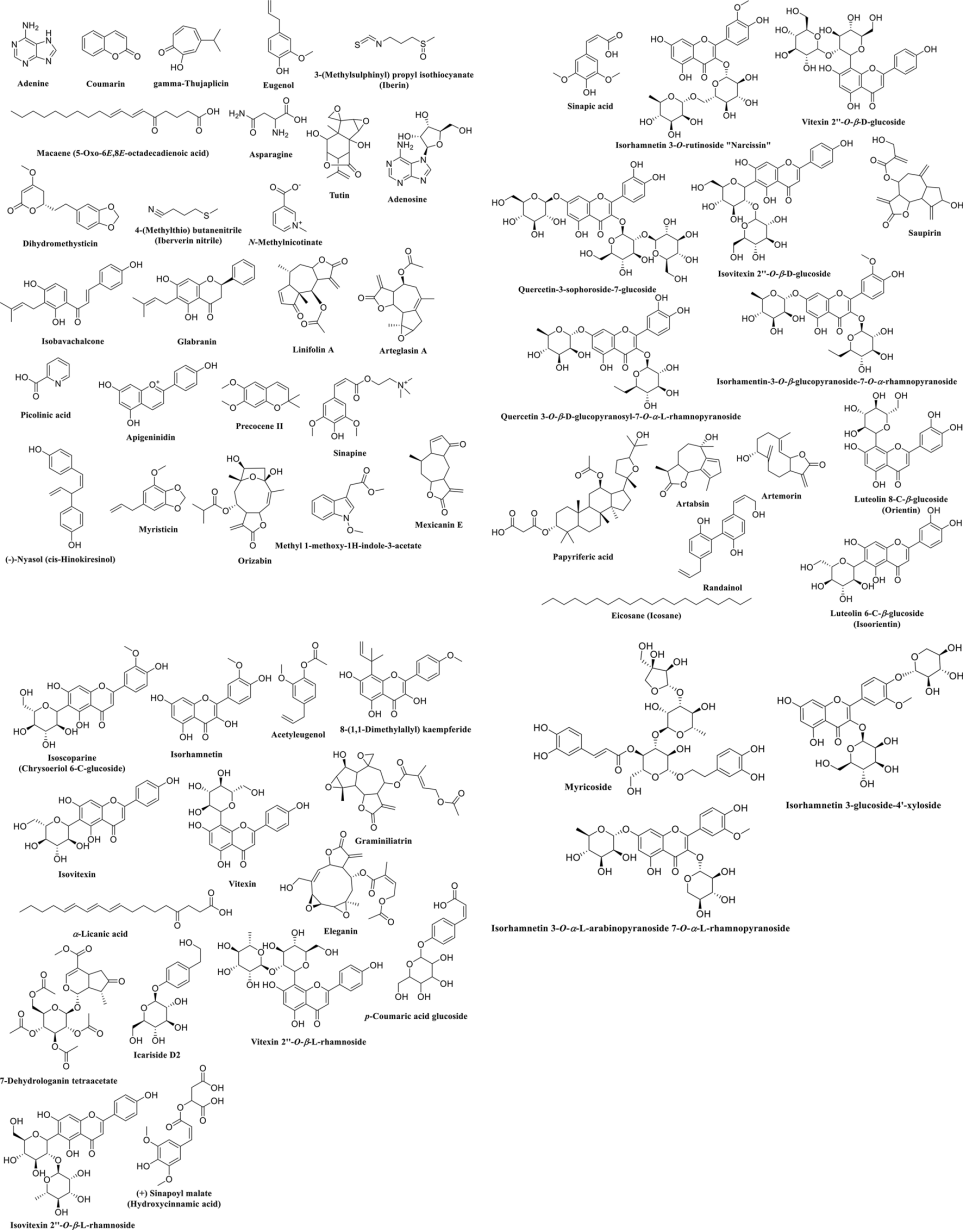

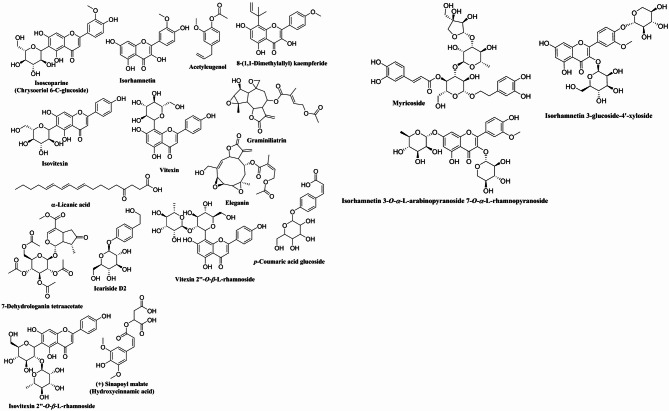
Structure of the identified metabolites in the defatted extract of *Malcolmia grandiflora* Kuntze.

Metabolic profiling of the defatted extract of *M. grandiflora* using UPLC-HRMS resulted in identification of 60 metabolites belonging to different chemical classes. This is the first report to provide metabolic profiling for *M. grandiflora*. The interpreted metabolites (Table 3; Figs. 1 and 2) could be classified according to their chemical groups as follows:

#### Flavonoids

Twenty flavonoids were identified from the mass ion peaks at *m/z* 324.10582, 255.07442, 788.20312, 594.15952, 610.15382, 624.17032, 448.10092, 462.11692, 316.05852, 610.15462, 432.10582, 368.11102, 578.15522, and 594.40082. The mass ion peak at *m/z* 324.10582 was in agreement with the molecular formula C_20_H_20_O_4_ and could be identified as isobavachalcone **(1a)** or glabranin **(1b)**. Isobavachalcone **(1a)** is a prenylated chalcone that was isolated from *Psoralea corylifolia* L.(Fabaceae)^20^, while glabranin **(1b)** was detected by UPLC-HRMS in the aerial parts of Calabrian *Glycyrrhiza glabra* (Fabaceae)^21^. From the MS/MS fragmentation; two signals were detected at *m/z* 69.0332 and *m/z* 97.0273 favor the presence of a prenyl side chain characteristic for isobavachalcone, in addition to the two fragments at *m/z* 127.0388 and *m/z* 163.0604 typical for chalcone nucleus, accordingly isobavachalcone is the best matching compound. The mass ion peak at *m/z* 255.07442 in agreement with the molecular formula C_15_H_11_O_4_^+^ was annotated as apigeninidin **(2)** an anthocyanidin which was isolated from the leaves of *Sorghum bicolor* (Poeaceae)^22^. The mass ion peak at *m/z* 788.20312 matched with the formula C_33_H_40_O_22_ and MS/MS fragmentation showed two key fragments at *m/z* 627.1608, noting the loss of one hexose, 465.1017 (loss of two hexoses), and 303.0501(characteristic for quercetin aglycon), accordingly it could be annotated as quercetin-3-sophoroside-7-glucoside **(3)** that was previously detected in *Brassica incana* (Brassicaeae) using HPLC-PDA-ESI-MS^23^. The mass ion peak at *m/z* 594.15952 perfectly matches the molecular formula C_27_H_30_O_15_ and exhibit MS/MS fragments at *m/z* 283.0601, 313.0709, 337.0708, that could be annotated as vitexin 2՝՝-*O*-*β*-D-glucoside **(4a)** or isovitexin 2՝՝-*O*-*β*-D-glucoside (saponarin) **(4b)**; both compounds **(4a)** and **(4b)** were identified in the petals of *Silene alba* (Caryophyllaceae)^24^. The mass ion peak at *m/z* 610.15382 agreed with the molecular formula C_27_H_30_O_16_ could be dereplicated as quercetin 3-*O*-*β*-D-glucopyranosyl-7-*O*-*α*-L-rhamnopyranoside **(5)** that was early isolated from the two Brassicaceae plants: *Arabidopsis thaliana* and *Erucaria hispanica*^25,26^. Similarly, the mass ion peak at *m/z* 624.17032 was in accordance with the molecular formula C_28_H_32_O_16_ could be annotated as isorhamentin-3-*O*-*β*-glucopyranoside-7-*O*-*α*-rhamnopyranoside **(6a)** or isorhamnetin 3-*O*-rutinoside (narcissin) **(6b).** Isorhamentin-3-*O*-*β*-glucopyranoside-7-*O*-*α*-rhamnopyranoside **(6a)** which was isolated from *Erucaria hispanica* (Brassicaceae)^27^, while isorhamnetin 3-*O*-rutinoside **(6b)** was detected in the aerial parts of *Sinapis pubescens* L. (Brassicaceae) *via* HPLC-PDA/ESIMS^28^. The mass ion peak at *m/z* 448.10092 matching the molecular formula C_21_H_20_O_11_ was identified as orientin; luteolin 8-C-*β*-glucoside **(7a)** or isoorientin; luteolin 6-C-*β*-glucoside **(7b)**; that were isolated from the Brassicaceae plants: *Morettia philaena* (Delile) DC. and *Erucaria hispanica*^27^. Signal at *m/z* 462.11692, in agreement with the molecular formula C_22_H_22_O_11_ was recognized as isoscoparine; chrysoeriol 6-C-glucoside (**8**) previously identified by HPLC-MS/MS of *Actinidia deliciosa* (Actinidiaceae)^29^. The molecular mass at *m/z* 316.05852 matching the molecular formula C_16_H_12_O_7_ was identified as isorhamnetin **(9)** that was formerly detected in the Brassicaceae plant *Sinapis arvensis*^30^, a related signal at *m/z* 610.15462 that was in perfect match with the molecular formula C_27_H_30_O_16_ (indicating flavonol diglycoside) together with its fragment at *m/z* 317.0653 (characteristic for isorhamnetin aglycon) was dereplicated as isorhamnetin 3-*O*-glucoside-4՝-*O*-xyloside **(10)** which was previously isolated from the non-polar extract of the Brassicaceae plant *Diplotaxis harra*^31^. The mass ion peak at *m/z* 432.10582, in perfect match with the molecular formula C_21_H_20_O_10__,_ together with MS/MS fragments at *m/z* 313.0714, 323.0925, 337.0699 and 367.0820 enabled the dereplication of this metabolite as vitexin **(11a)** or isovitexin **(11b).** Vitexin **(11a)** was early isolated from *Erucaria hispanica* (Brassicaceae)^27^, and isovitexin **(11b)** was identified in *Boreava orientalis* (Brassicaceae)^32^. The signal at *m/z* 368.11102 corresponding to the molecular formula C_21_H_20_O_6_ could be identified as 8-(1,1-Dimethylallyl) kaempferide **(12)** which was detected in *Kaempferia galanga* (Zingiberaceae)^33^, another mass ion peak at *m/z* 578.15522 agreed with the molecular formula C_27_H_30_O_14_ was annotated as vitexin 2՝՝-*O*-rhamnoside **(13a)** or isovitexin 2՝՝-*O*-rhamnoside **(13b).** Vitexin 2՝՝-*O*-rhamnoside (**13a)** was detected in *Crataegus monogyna* leaves (Rosaceae)^34^, and isovitexin 2՝՝-*O*-rhamnoside **(13b)** was isolated from *Mimosa xanthocentra* Mart. (Leguminosae)^35^. A further mass ion peak at *m/z* 594.40082 matched with the molecular formula C_27_H_30_O_15_ could be identified as isorhamnetin 3-*O*-*α*-L-rhamnopyranosyl-(1→2)-*α*-L-arabinopyranoside **(14)** previously isolated from *Taverniera aegyptiaca* (Fabaceae)^36^.

#### Phenolics

Seven phenolics were tentatively identified based on the mass to charge ratios (*m/z*) of 146.03672, 220.12092, 224.13162, 756.21482, 300.06862, 326.07072, and 340.14592. The mass ion peak at *m/z* 146.03672 was in agreement with the molecular formula C_9_H_6_O_2_ and could be identified as coumarin **(15)** which was detected in Brassicaceae plants using phytochemical screening^37^, other mass ion peak at *m/z* 220.12092 matching with the molecular formula C_13_H_16_O_3_ could be annotated as precocene II **(16)** a characteristic chromene (benzopyran) widely distributed in family Asteraceae^38^, another mass ion peak at *m/z* 224.13162 was in agreement with the molecular formula C_11_H_12_O_5_ and dereplicated as sinapic acid **(17)** which was detected before in *Arabidopsis thaliana* and *Lepidium sativum* (Brassicaceae)^25,26^, another mass ion peak at *m/z* 756.21482 was in agreement with the molecular formula C_33_H_40_O_20_ and dereplicated as myricoside **(18)** a phenylethanoid which was detected before in the roots of *Clerodendrum myricoides* (Verbenaceae)^39^, further mass ion peak at *m/z* 300.06862 in accordance with the molecular formula C_14_H_20_O_7_ could be annotated as icariside D2 **(19)** previously isolated from *Brassica oleracea* var. *acephala* (Brassicaceae)^40^, additional mass ion peak at *m/z* 326.07072 in agreement with the molecular formula C_15_H_18_O_8_ could be identified as *p*-coumaric acid glucoside **(20)** and was isolated before from *Lobularia maritima* (Brassicaceae)^41^, finally the mass ion peak at *m/z* 340.14592 corresponding to the molecular formula C_15_H_16_O_9_ was annotated as (+) sinapoyl malate **(21)** a sinapic acid derivative that was previously detected in *Lepidium sativum* and *Brassica incana* (Brassicaceae)^23,25^.

#### Sesquiterpenoids

Ten sesquiterpenoids were identified from the signals at *m/z*: 294.10632, 304.12712, 366.14312, 232.12142, 346.11702, 248.15282, and 434.16052. Firstly, the mass ion peak at *m/z* 294.10632 corresponding to the molecular formula C_15_H_18_O_6_ was tentatively annotated as tutin **(22)** previously isolated from *Coriaria japonica* (Coriariaceae)^42^, other signal at *m/z* 304.12712 (C_17_H_20_O_5_) was identified as linifolin A **(23a)** or arteglasin A **(23b)**, linifolin A **(23a)** was early isolated from the *Helenium* species (Asteraceace)^43^, while arteglasin A **(23b)** was previously isolated from another Asteraceae plant *Chrysanthemum indicum*^44^. Another mass ion peak at *m/z* 366.14312 (C_19_H_26_O_7_) could be annotated as orizabin **(24)** which was isolated previously from *Ipomoea orizabensis* (Convolvulaceae)^45^, additional mass ion peak at *m/z* 232.12142 (C_14_H_16_O_3_) was identified as mexicanin E **(25)** which is a norsesquiterpenoid lactone previously reported in *Helenium microcephalum* (Asteraceae)^46^, furthermore the mass ion peak at *m/z* 346.11702 (C_19_H_22_O_6_) was tentatively identified as saupirin **(26)** that was isolated from *Acroptilon repens* (Asteraceae)^47^, moreover the mass ion peak at *m/z* 248.15282 (C_15_H_20_O_3_) could be identified as artabsin **(27a)** or artemorin **(27b)** that were previously detected in the genus *Artemisia* (Asteraceae)^48,49^, finally, the mass ion peak at *m/z* 434.16052 (C_22_H_26_O_9_) could be annotated as graminiliatrin **(28a)** or eleganin **(28b)** which were isolated from *Liatris* species (Asteraceae)^50^.

#### Nitrogenous compounds

Nine nitrogenous compounds were putatively identified from the mass ion peaks at *m/z*: 135.06792, 132.05302, 267.09562, 137.04702, 123.03132, 309.12112, 186.02942, and 219.08992. The mass ion peak at *m/z* 135.06792 (C_5_H_5_N_5_) was tentatively identified as adenine **(29)** a compound previously detected in *Brassica oleracea* var. *acephala* (Brassicaceae)^40^, other mass ion peak at *m/z* 132.05302 (C_4_H_8_N_2_O_3_) was identified as asparagine **(30)** an alpha amino acid distributed in family Brassicaceae^51^, another mass ion peak at *m/z* 267.09562 (C_10_H_13_N_5_O_4_) was annotated as adenosine **(31)** a nucleoside detected early in *Arabidopsis thaliana* and *Brassica oleracea* var. *acephala* (Brassicaceae)^25,40^, additional mass ion peak at *m/z* 137.04702 (C_7_H_7_NO_2_) was recognized as *N*-methylnicotinate (trigonelline) **(32)** a compound that was detected before in *Oryza sativa* (Poaceae) and *Lotu japonicus* (Fabaceae)^52^, moreover the molecular ion peak at *m/z* 123.03132 (C_6_H_5_NO_2_) was identified as picolinic acid **(33)** an intermediate in tryptophane metabolism^53^, further mass ion peak at *m/z* 309.12112 (C_16_H_24_NO_5_^+^) was putatively identified as sinapine (sinapoylcholine) **(34)** that was detected previously in *Arabidopsis thaliana* and *Lepidium sativum* (Brassicaceae)^25,26^, furthermore the mass ion peak at *m/z* 186.02942 (C_11_H_10_N_2_O) could be recognized as caulilexin C (1-methoxyindole-3-acetonitrile) **(35a)**, or arvelexin (4-methoxyindole-3-acetonitrile) **(35b)**; both compounds were early detected in *Brassica oleracea* var. *acephala* (Brassicaceae)^40^, finally, the signal at *m/z* 219.08992 (C_12_H_13_NO_3_), was annotated as methyl-1-methoxy-1H-indole-3-acetate **(36)** that was also detected in *Brassica oleracea* var. *acephala* (Brassicaceae)^40^.

#### Terpenoids

Four essential oil compounds were putatively identified from the mass ion peaks at *m/z*: 164.08372, 192.09012, and 206.09812. The molecular ion peak at *m/z* 164.08372 (C_10_H_12_O_2_) was recognized as gamma-thujaplicin **(37a)** that was isolated from *Thujopsis dolabrata* (Cupressaceae)^54^, and eugenol **(37b)** detected in *Aurinia sinuata* (Brassicaceae) by GC-MS analysis^55^, another mass ion peak at *m/z* 192.09012 (C_11_H_12_O_3_) could be identified as myristicin **(38)** which was detected by GC-MS analysis of *Nasturtium officinale* (Brassicaceae) essential oil^56^, and finally signal at *m/z* 206.09812 (C_12_H_14_O_3_) annotated as acetyleugenol **(39)** an oil component previously detected in *Syzygium aromaticum* L. (Myrtaceae)^57^.

#### Isothiocyanate compounds

Two isothiocyanate compounds were identified from the mass ion peaks at *m/z*: 163.10302 and 115.06362 corresponding to the molecular formulas C_5_H_9_NOS_2_ and C_5_H_9_NS annotated as 3-(methylsulphinyl) propyl isothiocyanate (iberin) **(40)** and 4-(methylthio) butanenitrile (iberverin nitrile) **(41)**, respectively; both were detected in *Lobularia libyca* (Brassicaceae) using GC-MS analysis^58^.

#### Other metabolites

Two fatty acids were identified from the mass ion peaks at *m/z* 294.15772 and 292.20382. They were matched with the molecular formulas C_18_H_30_O_3_ and C_18_H_28_O_3_ and identified as macaene (5-Oxo-6*E*,8*E*-octadecadienoic acid) **(42)** and *α*-Licanic acid **(43); 42** was early detected in *Lepidium meyenii* ‘maca’ (Brassiaceae)^59^ and **43** in *Acioa edulis* (Prance) seeds (Chrysobalanaceae)^60^.

Two lignan compounds were also identified from the mass ion peaks at *m/z* 252.11122 and 282.13732 compatible with the molecular formulas C_17_H_16_O_2_ and C_18_H_18_O_3_, respectively, and could be dereplicated as (-)-nyasol (cis-Hinokiresinol) **(44)** and randainol **(45)**. (-)-nyasol **(44)** a norneolignan previously isolated from the rhizomes of *Anemarrhena asphodeloides* (Asparagaceae)^61^, and randainol **(45)** a neolignan isolated from *Sassafras randaiense* (Lauraceae)^62^.

One triterpene identified as papyriferic acid **(46)** from the mass ion peak at *m/z* 604.35142 (C_35_H_56_O_8_), was previously isolated from *Betula neoalaskana* (Betulaceae)^63^. One phytolactone with the molecular formula C_15_H_16_O_5_ that matched the mass ion peak at *m/z* 276.09592 was identified as dihydromethysticin **(47)** a phytolactone formerly detected in *Piper methysticum* (Piperaceae) by LC-HRMS^64^. One alkane identified as eicosane (icosane) **(48)** was annotated from the mass ion peak at *m/z* 282.13732 and the molecular formula C_20_H_42_, this was identified in *Arabidopsis thaliana* (Brassicaceae)^25^. Finally, one iridoid glycoside was identified as 7-dehydrologanin tetraacetate **(49)** from the mass ion peak at *m/z* 556.17272 and the molecular formula C_25_H_32_O_14_^65^.

### Antioxidant capacity of *M. grandiflora* Kuntze

The antioxidant capacity of *M. grandiflora* implies the plant’s ability to quench free radicals and reactive oxygen species, primarily due to its phenolic and flavonoid constituents. The antioxidant activity of the *n*-hexane and defatted extracts was evaluated adopting DPPH, ABTS and FRAP assays. The results of DPPH and ABTS were expressed as IC_50__,_ while FRAP was expressed as ascorbic acid equivalent/g dry extract (Table 4).

The antioxidant evaluation of *M. grandiflora* revealed distinct differences between the extracts across the applied assays. The defatted extract demonstrated notable ABTS scavenging activity, exhibiting an IC₅₀ value of 25.67 µg/mL, whereas its activity against DPPH was markedly weaker, with an IC₅₀ of 835.23 µg/mL compared with ascorbic acid. Similarly, the *n*‑hexane extract displayed measurable antioxidant potential in the ABTS assay with an IC₅₀ of 36.44 µg/mL; however, it did not produce significant DPPH scavenging effects at concentrations up to 1000 µg/mL.

The FRAP assay further supported the antioxidant potential of the extracts, with the defatted and *n*‑hexane fractions exhibiting reducing capacities of 1.76 and 1.41 mmol AA/g extract, respectively. These findings collectively indicate that the extracts possess differential reactivity toward various free radical species, likely reflecting differences in their phytochemical constituents. As in DPPH and ABTS assays the evaluation of the antioxidant activity depends on the free radicals neutralizing efficacy of the extract constituents leading to concentration dependent discoloration of the purple color of DPPH solution and the blue color of ABTS reagents. A large decrease in the absorbance of the reaction mixture indicates significant free radical scavenging activity of the extract. In contrast, FRAP assay depends on the ability of the antioxidant constituent to reduce ferric ion in the reagent to ferrous forming blue complex so the increase in the absorbance indicates strong antioxidant^66^. Based on the current findings, ABTS and FRAP assays showed good antioxidant activity for the defatted extract than the *n*-hexane extract while DPPH showed weak activity of both tested extracts. Previous report on the antioxidant activity of *M. africana* was measured by DPPH, ABTS and *β*-carotene linoleic acid assays, results revealed that the IC_50_ 39.5 ± 0.005 µg/mL of the methanolic extract of the aerial parts showed the strongest antioxidant capacity using DPPH, while the methanolic extract of the seeds and aerial parts inhibit linoleic acid oxidation; this makes the methanolic extract good preservative for food and pharmaceutical preparations^14^. Another report on antioxidant potential of *M. aegyptica* used in vitro assays as DPPH, hydrogen peroxide capacity, hydroxyl radical (HO•) scavenging capacity and hemolysis, and in vivo assay as TBARS test (thiobarbituric acid reactive substances) the results revealed that the methanolic extract of *M. aegyptica* possessed good antioxidant capacity^7^.

### Anti-inflammatory potential of *M. grandiflora* Kuntze

The anti-inflammatory activity of the *n*-hexane and defatted extracts was evaluated through in vitro inhibition of COX-1 and COX-2. Results (Table 5) implied that the *n*-hexane extract of *M. grandiflora* inhibits COX-1 and COX-2 enzymes with IC_50_ values of (5.208 ± 0.220 and 3.391 ± 0.150 µg/mL, respectively, SI = 1.54) compared to ibuprofen (6.195 ± 0.23 and 2.398 ± 0.09 µg/mL, respectively, SI = 2.58), while the defatted extract exhibited potent selectivity against COX-2 inhibition activity (IC_50_ = 1.723 ± 0.060 µg/mL, SI = 5.02) compared to the standard inhibitor celecoxib (IC_50_ = 0.786 ± 0.030 µg/mL, SI = 27.40). The selectivity index (SI) is calculated as the ratio of IC₅₀ for COX-1 to IC₅₀ for COX-2. An SI value greater than 1 indicates preferential inhibition of COX-2, which is associated with a lower risk of gastric ulceration compared to the non-selective COX inhibitors. In this study, the *n*-hexane and the defatted extracts exhibited SI values of 1.54 and 5.02, respectively. These findings demonstrate that both extracts act as selective COX-2 inhibitors, with the defatted extract showing markedly higher selectivity than the *n*-hexane extract. A review of existing literature revealed a prior in vivo study by Chouikh et al. (2024) that assessed the anti-inflammatory activity of *M. aegyptiaca* in rats using hot-plate, writhing, and carrageenan-induced paw edema assays. The results of that study demonstrated that the methanolic extracts of *M. aegyptiaca* possessed analgesic and anti-inflammatory properties, thus supporting its traditional application in managing pain and inflammation^7^.

### Cytotoxic activity of *M. grandiflora* Kuntze

The cytotoxic activity of the *n*-hexane and defatted extracts was assessed on two cell lines MCF-7 and Caco-2. The results in (Table 5) showed that the *n*-hexane and the defatted methanol extract were exhibited significant inhibitory activity against MCF-7 cell line with IC_50_ = 3.855 ± 0.120 µg/mL and 7.271 ± 0.420 µg/mL, respectively, which is lower than that of staurosporine with IC_50_ = 13.630 ± 0.660 µg/mL. While the results of the *n*-hexane and defatted extracts against Caco-2 cell line showed inhibitory effect with IC_50_ = 2.892 ± 0.110 µg/mL and 15.070 ± 0.820 µg/mL, respectively compared to that of staurosporine with IC_50_ = 2.016 ± 0.098 µg/mL. Prior research on *M. littorea* (L.) R.Br. extract against HepG2 (human liver carcinoma) cells and HEK 293 cells (Human embryonic kidney 293 cells) revealed strong tyrosinase inhibitory activity and reduction in the viability of HepG2 cells and showed lower toxicity towards HEK 293 cells^67^.

### Molecular docking studies

Cyclin dependent kinases (CDKs) are a family of enzymes that play crucial roles in regulating the progression of the cell through different stages of the cell cycle^68^. Each CDK isozyme is responsible for specific functions within the cell cycle^69^. Overactivation of certain CDK enzymes has been reported in various cancers^70^, therefore members of the CDK family are valuable targets for cancer management therapy. Overactivation of CDK2 and its related subunits is a recurrent oncogenic event that has been reported in various malignancies^71^, similarly CDK4 and CDK6 aberrant activation is a key feature in several cancer types particularly breast cancer^72,73^.

**Fig. 3:**
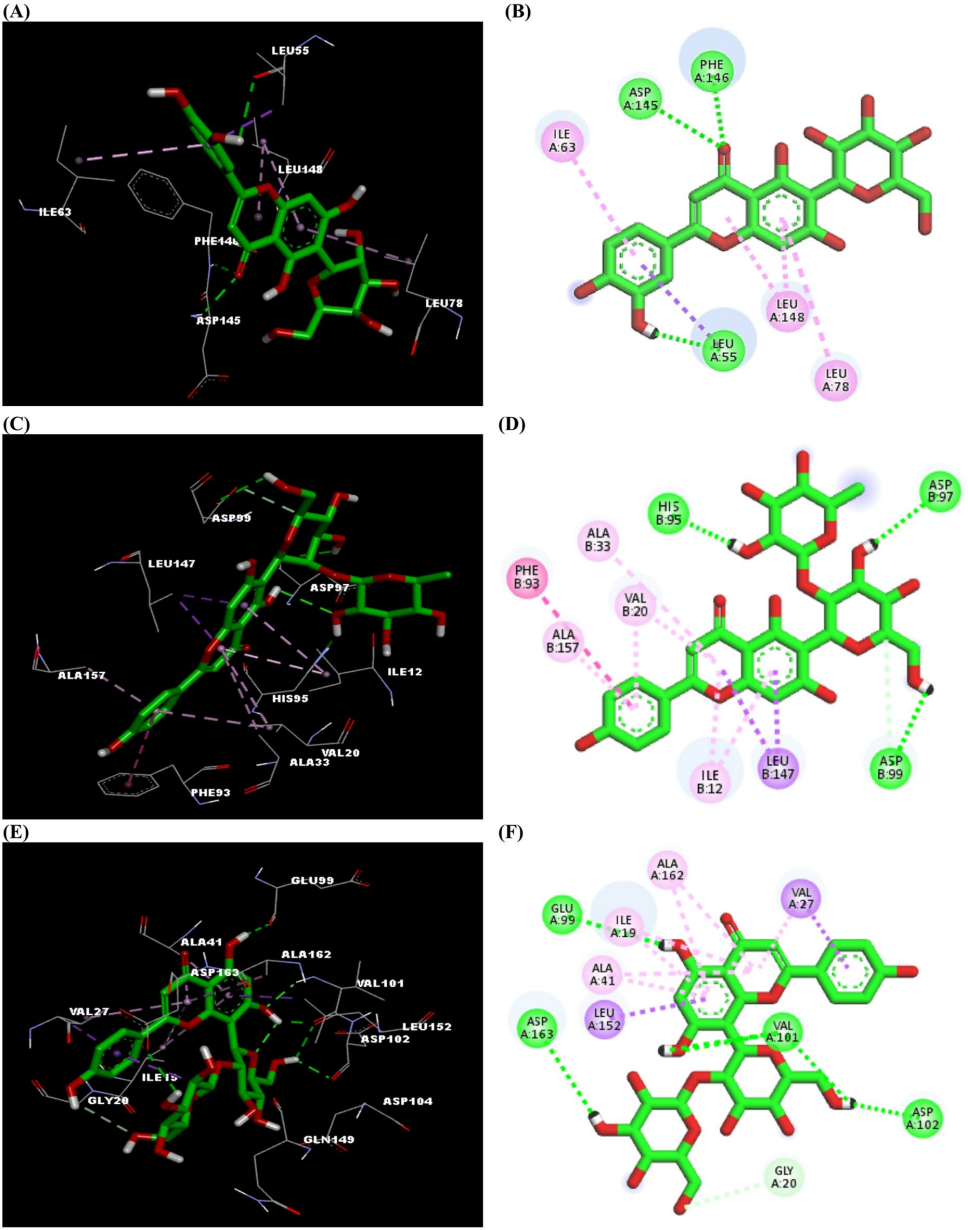
3D and 2D interactions of CDK2 with isoorientin (A and B), CDK4 with isovitexin-2՝՝-*O*-rhamnoside (B and C), and CDK6 with vitexin 2՝՝-*O*-*β*-D-glucoside (E and F), respectively. Ligand is represented by green stick model. Residues are depicted in line models in 3D and circles in 2D model. Hydrogen bond (green), *π*-alkyl (pink), and *π*-sigma (magenta) interactions are depicted in dashed lines.

Metabolites tentatively identified *via* both GC-MS and UPLC-HRMS analyses of the *n*-hexane and defatted extracts of *M. grandiflora*, respectively, were subjected to molecular docking studies to provide insight into their potential to inhibit certain members of CDK family (CDK2, CDK4, and CDK6). PDB entries for the three enzymes (7RXO, 9CSK, and 9D8U, respectively) have been selected based on certain parameters including source (homo sapiens), high resolution, X-ray diffraction method, and to be recently deposited^74^. The binding sites of the CDK enzymes in the current work have been selected based on the interactions of the co-crystallized ligands. Interestingly, examination of the docking results of the metabolites detected in both extracts declared differences in their efficacy to modulate CDKs activities. Non-polar metabolites composed mainly of hydrocarbons, fatty acids and esters, detected in *M. grandiflora*, displayed moderate binding affinities towards the three CDK isozymes expressed as docking scores ranging from − 4.8 to −8.8 kcal/mol compared to those for the co-crystallized ligand of CDK2 (WN333, −10.2 kcal/mol) and that of CDK4 and CDK6 (atirmociclib, −9.8 and − 9.2 kcal/mol, respectively) (Table 6). While on the other side, most of the metabolites in the defatted extract, especially flavonoids, exhibited strong binding affinities with CDKs that, in some cases, surpassed those of the co-crystalized ligands. Considering the variable classes detected in the UPLC-MS analysis of the defatted extract, flavonoids were found to be the most potent inhibitors of the CDKs, where isoorientin along with vitexin and isovotexin (and their related derivatives) exhibited strong binding scores with the three CDKs. Moreover, the sesquiterpenoids linifolin A and orizabin displayed characteristic selectivity profile towards CDK4 over other tested CDKs. Importantly, selectivity for particular CDKs is vital in preclinical development, since it helps maximize therapeutic efficacy while minimizing toxicity^75^. The flavonoid isoorientin exhibited comparable CDK2 binding affinity (−10.3 kcal/mol) to that of the co-crystallized ligand “WN333” (−10.2 kcal/mol), where it occupied the allosteric pocket in the enzyme with its carbonyl oxygen forming two hydrogen bonds with Asp145 and Phe146, two residues of the highly conserved DFG (Asp-Phe-Gly) motif in the enzyme, and an additional hydrogen bonding connecting its OH-C3՝ with the C-helix residue; Leu55, besides two π-alkyl bonds between ring A and the amino acids; Leu78 and Leu148. Finally, ring B formed π-alkyl and π-sigma bonds with Ile63 and Leu55, respectively (Fig. 3A and B). Isoorientin has been reported previously to inhibit various cancer cell lines including colorectal, gastric, and lung cancers and to reduce expression levels of CDK1, CDK2, and CDK6 in treated cells^76–78^.

On the other hand, isovitexin 2՝՝-*O*-rhamnoside unveiled CDK4 binding affinity (−9.8 kcal/mol) that is equal to that of the co-crystallized ligand atirmociclib, where it anchored in ATP-binding site forming three hydrogen bonds with hinge residues; His95, Asp97, and Asp99, respectively and two *π*-sigma bonds with Leu147. Ring B engaged in *π*-*π* stacking with Phe93. Additionally, the three rings of the flavonoid nucleus were involved in six π-alkyl interactions with Ile12, Val20, Ala33, and Ala157 (Fig. 3C and D).

For CDK6, the highest binding affinity exhibited by vitexin 2՝՝-*O*-*β*-D-glucoside (−9.6 kcal/mol) exceeded that of atirmociclib (−9.2 kcal/mol) where it bound through three hydrogen bonds to Val101, a previously reported residue as an important target to various inhibitors of the enzyme^79^ and three more hydrogen bonds with Glu99, Asp102, and Asp163, respectively. Rings A and B of the flavonoid nucleus were involved in *π*-sigma bonds with Leu152 and Val27, respectively, while both rings A and C were incorporated in seven π-alkyl interactions with four amino acids: Ile19, Val27, Ala41, and Ala162 (Fig. 3E and F).

Viexin and isovitexin have been previously reported for their anticancer potential against certain cancer cell lines including lymphoma and colon cancer^80,81^. Their binding affinities to CDK2, CDK4, and CDK6 have also been demonstrated through molecular docking studies^82^. However, to the best of our knowledge, this is the first docking study investigating isovitexin 2՝՝-*O*-rhamnoside and vitexin 2՝՝-*O*-*β*-D-glucoside against CDKs and exploring their molecular interactions. These results highlight the broad spectrum CDK inhibition of phenolics and flavonoids, while on the other hand terpenoids may yield subtype selectivity.

Analysis of the docking results in conjunction with the biological testing outcomes indicated a discrepancy in behavior. The *n*-hexane fraction showed significant in vitro cytotoxicity towards both Caco-2 and MCF-7 cell lines, that is greater than the efficacy of the defatted extract, whereas theoretical results of the docking simulation studies recommend a higher CDK inhibition of the polar metabolites identified in the defatted extract. These observations may indicate that the cytotoxic activity of nonpolar metabolites is mediated through molecular mechanisms other than CDK inhibition. Conversely, the polar metabolites, despite their lower cytotoxicity in their crude form, may operate *via* more selective molecular pathways, as evidenced by their predicted binding affinities for CDKs. Our comprehensive docking analysis highlights a number of metabolites with certain selectivity profiles, suggesting further exploration for the development of potent targeted anticancer agents.

## Experimental section

General instruments and chemicals are provided in the Supplementary material.

### Plant material

*Malcolmia grandiflora* Kuntze (family Brassicaceae); aerial parts were collected from the northern region of Saudi Arabia (30°15’27.7"N, 42°27’23.5"E), in March 2023. The plant identity was confirmed by Mr. Abdulatif M. Al-Othaim, a local botanical expert. A specimen designated as QPP-120 was kept at college of pharmacy, Qassim university, Saudi Arabia. The aerial part of the plant was cleaned, dried, then powdered. The powdered plant material (500 g) was extracted using aqueous methanol (80%, 4 × 1000 mL). The filtered extracts were pooled and concentrated under vacuum at a temperature not exceeding 50 °C using rotatory evaporator. The concentrated methanol extract was then defatted using *n*-hexane (5300 mL), the *n*-hexane extract was evaporated under vacuum till dryness. As well the defatted extract was evaporated under vacuum till dryness; both fractions were kept in umber light vials at 5° till further use.

### Total phenolic content (TPC)

Folin–Ciocalteu method was used for determination of the total phenolic content (TPC) of the alcoholic extract by the method^83^. Calibration curve of gallic acid was plotted and TPC was expressed as mg gallic acid equivalent/gram dry extract. The full method is provided in the supplementary material.

### Total flavonoid content (TFC)

Aluminum chloride was used for determination of TFC content in the alcoholic extract according to the method reported by^84^. The calibration curve of quercetin was plotted, TFC was expressed as mg of quercetin/gram dry extract. The full method is provided in the supplementary material.

### GC-MS analysis

GC-MS analysis was conducted for the *n*-hexane extract on a Shimadzu GC/MS-QP 2010 with a DB-5 column. The oven was programmed from 50 to 300 °C at 5 °C/min, helium was used as carrier gas (1.37 mL/min), and samples (1% v/v, 2 µL) were injected in split mode (15:1). EI ionization (70 eV) was applied, scanning *m/z* 35–500. Identification of the chemical constituents was performed by cross-referencing their retention times and mass fragmentation patterns with the Wiley and National Institute of Standards and Technology (NIST) mass spectral library databases.

### LC-HRMS analysis

Compounds occurred in the defatted extract were separated on a Thermo Scientific C18 column (3 × 150 mm, 3 μm) using an UltiMate 3000 UHPLC with a 22 min gradient (0.1% formic acid in water [A] and acetonitrile [B]) at 0.4 mL/min and 40 °C. The gradient progressed from 5% to 80% B in 15 min, then returned to 5% B. Injection volume was 3 µL. HRMS was performed on a Bruker MicroTOF QIII with positive ESI (capillary 4500 V, nebulizer 2.0 bar, drying gas 8 L/min at 300 °C), scanning *m/z* 50–1000. Data were processed using Compass Data Analysis software^85,86^.

### In vitro biological assays

#### Antioxidant assays

The antioxidant activity of the *n*-hexane and defatted extracts were assessed by DPPH, ABTS and FRAP^87–89^. Detailed protocols were provided in the supplementary material.

#### Anti-inflammatory activity

The ability of the *n*-hexane and defatted extracts to inhibit COX-1 and COX-2 enzymes was tested and compared with known inhibitors such as celecoxib and indomethacin, which served as positive controls. This assay used commercially available enzyme immunoassay kits designed for human COX-1 and COX-2 inhibition screening, and the procedures followed the manufacturer’s instructions (catalog numbers 701070 and 701080 from Cayman Chemical)^90,91^. The full method is provided in the supplementary material.

#### Cytotoxic activity

The cytotoxic activity of the *n*-hexane and defatted extracts were examined using a cell viability assay, following a method previously described by Mosmann in 1983^92,93^. Two cancer cell lines MCF-7 (breast carcinoma) and Caco-2 (colon adenocarcinoma) obtained from the American Type Culture Collection (ATCC) that was supplied by Vacsera, Egypt, were used for the experiment. The full method is provided in the supplementary material.

### Statistical analysis

The experiments in this study were conducted in triplicate, and the data were presented as means ± standard errors of the means (SEM). Statistical significance was determined using one-way ANOVA followed by the Tukey’s post hoc test for multiple comparisons.

### Molecular docking

Compounds annotated during GC-MS and UPLC-HRMS analyses of the *n*-hexane and defatted extracts of *M. grandiflora*, respectively were docked into the active sites of CDK2, CDK4, and CDK6 enzymes using PDB entries: 7RXO^71^, 9CSK^72^, and 9D8U^73^, respectively that were downloaded from Protein Data Bank (https://www.rcsb.org/). Structures of compounds were retrieved from PubChem^94^[August, 2025]. Docking study was performed using Autodock Vina *via* Pyrx platform^95^. XYZ coordinates were set as; 7RXO: −20.25, −2.12, −19.54; 9CSK: 8.43, 0.23, 33.60; 9D8U: 16.96, 27.03, 8.47. Visualization and analysis of the docked poses were accomplished using Pymol software^96^ and BIOVIA Discovery Studio visualizer v21.1.0.20298 (Dassault systems Biovia Corp., San Diego, CA, USA).

## Conclusion

The present study provides the first comprehensive phytochemical and bioactivity profiling of *M. grandiflora* Kuntze using GC‑MS and UPLC‑MS/MS analyses across non‑polar and polar fractions, thereby contributing novel insights into the chemical diversity and therapeutic potential of this understudied species. The identification of an extensive array of metabolites belonging to variable chemical classes such as flavonoids, phenolic derivatives, and sesquiterpenoids which represents a significant advancement in the phytochemical characterization of the genus. A major finding of this work is the clear correlation between the cytotoxic activity of the *n*‑hexane extract and its distinctive metabolic constituents, positioning this fraction as a promising reservoir of broad-spectrum cytotoxic agents. In addition, the defatted polar extract demonstrated a repository of phenolic and flavonoid compounds, whereas in silico molecular interactions reveal a selective inhibitory affinity toward cyclin-dependent kinases (CDKs), highlighting their potential relevance in targeted anticancer strategies.

Notably, the flavonoids isoorientin, isovitexin 2՝՝‑*O*‑rhamnoside, and vitexin 2՝՝‑*O*‑*β*‑D‑glucoside, along with the sesquiterpenoids linifolin A and orizabin, emerged as key bioactive candidates with mechanistic implications in CDK modulation. These findings underscore the novelty of this investigation in linking specific metabolite classes to distinct cytotoxic and enzyme‑inhibitory profiles within *M. grandiflora*. According to these outcomes, future research should focus on the isolation, structural elucidation, and mechanistic evaluation of the highlighted compounds, particularly through targeted CDK inhibition assays and broader anticancer screenings.

**Table 1:** Total phenolic and flavonoid contents of *M. grandiflora* Kuntze alcoholic extract.

	TPC (mg GAE/g)^a^	TFC (mg QE/g)^b^
*M. grandiflora* Kuntze alcoholic extract	81.059 ± 5.76	17.23 ± 0.454

^a^ mg gallic acid equivalent in 1 g of dry extract, ^b^ mg quercetin equivalent in 1 g of dry extract.

TPC: Total phenolic content, TFC: Total flavonoid content.

**Table 2:** GC-MS analysis results of the *n*-hexane extract of *M. grandiflora* Kuntze.

	Rt	Identified compounds	Molecular formula	Area %
1.	31.820	1,7-Octadiene, 2,5-bis-(cis)-(2,2-dimethyl-3-carboxycyclopropyl)	C_20_H_30_O_4_	3.13
2.	33.590	Hexadecanoic acid, methyl ester (Palmitic acid, methyl ester)	C_17_H_34_O_2_	7.07
3.	36.800	9-Tetradecynoic acid, methyl ester or (+)-(*Z*)-Longipinane)	C_15_H_26_O_2_	4.21
4.	36.875	9,12,15-Octadecatrienoic acid, methyl ester (Linolenic acid, methyl ester)	C_19_H_32_O_2_	17.77
5.	37.335	2-Hexadecen-1-ol, 3,7,11,15-tetramethyl-, acetate, [R-[R*,R*-(*E*)]]	C_22_H_42_O_2_	21.01
6.	41.195	13-Docosenamide, (*Z*)	C_22_H_43_NO	10.33
7.	43.965	Cyclononasiloxane, octadecamethyl	C_18_H_54_O_9_Si_9_	1.45
8.	46.305	(3R)−3-Ferrocenyl-3,4-dihydroisoquinoline	C_19_H_17_FeN	2.16
9.	48.500	Ethyl 9, 12, 15 -octadecatrienoate	C_20_H_34_O_2_	2.48
10.	50.555	Hexadecanedioic acid	C_16_H_30_O_4_	2.20
11.	52.700	1-Chloro-5-nitroanthraquinone	C_14_H_6_ClNO_4_	2.17
12.	54.205	Tricyclo[5.3.1.0(6,11)]undecane-11-carboxylic acid, 1,5,5-trimethyl-8-oxo	C_15_H_22_O_3_	0.89
13.	54.360	Methyl 3-cis,9-cis,12-cis-octadecatrienoate	C_19_H_32_O_2_	1.83
14.	55.763	Tetraacetyl-*d*-xylonic nitrile	C_14_H_19_NO_9_	11.26
15.	56.750	Cis-vaccenic acid (cis-11-Octadecenoic acid)	C_18_H_34_O_2_	1.80
16.	57.025	Benzene, (1-hexyloctyl)-	C_20_H_34_	4.80
17.	57.075	Cyclotrisiloxane, hexamethyl	C_6_H_18_O_3_Si_3_	1.35
18.	58.675	Octahydrobenzo[*β*]pyran,4*α*-acetoxy-5,5,8*α*,-trimethyl	C_14_H_24_O_3_	0.88
19.	61.485	5-Benzofuranacetic acid,6-ethenyl − 2,4,5,6,7,7*α*-hexahydro-3,6-dime	C_16_H_20_O_4_	0.80
20.	62.845	Phen-1,4-diol, 2,3-dimethyl-5-trifluoromethyl	C_9_H_9_F_3_O_2_	0.60

**Table 3:** Putatively identified metabolites in the defatted extract of *M. grandiflora* Kuntze using LC-HRMS analysis.

*No.*	Tentative identification	Rt	m/z	M + H	Formula	MS/MS-Fragments	References
Flavonoid							
1.	**1a-** Isobavachalcone	1.9	324.10582	325.1131	C_20_H_20_O_4_	69.0332 97.0273 127.0388 163.0604	^16^
	**1b-** Glabranin (8-prenylpinocembrin)						^17^
2.	Apigeninidin	3.2	255.07442	256.0817	C_15_H_11_O_4_^+^	97.0268	^18^
3.	Quercetin-3-sophoroside-7-glucoside	6.9	788.20312	789.2104	C_33_H_40_O_22_	303.0501 465.1017 627.1608	^19^
4.	**4a**- Vitexin 2՝՝-*O*-*β*-D-glucoside	7.2	594.15952	595.1668	C_27_H_30_O_15_	283.0601 313.0709 337.0708 397.0921 595.1676	^20^
	**4b**- Isovitexin 2՝՝-*O*-*β*-D-glucoside (saponarin)						^20^
5.	Quercetin 3-*O*-*β*-D-glucopyranosyl-7-*O*-α-L-rhamnopyranoside	7.2	610.15382	611.1611	C_27_H_30_O_16_	299.0546 329.0658 330.0681 339.0881 413.0888	^21,22^
6.	**6a-**Isorhamentin-3-*O*-*β*-glucopyranoside-7-*O*-*α*-rhamnopyranoside	7.3	624.17032	625.1776	C_28_H_32_O_16_	303.0507 313.0697 343.0809 367.0861 625.1765	^23^
	**6b-** Isorhamnetin 3-*O*-rutinoside (Narcissin)						^24^
7.	**7a-** Luteolin 8-C-*β*-glucoside (Orientin)	8.0	448.10092	449.1082	C_21_H_20_O_11_	299.0560 300.0606 311.0536 329.0655 431.0979	^23^
	**7b-** Luteolin 6-C-*β*-glucoside (Isoorientin)						^23^
8.	Isoscoparine (Chrysoeriol 6-C-glucoside)	8.3	462.11692	463.1242	C_22_H_22_O_11_	298.0458 313.0703 314.0750 367.0804 445.1091	^25^
9.	Isorhamnetin	8.3	316.05852	317.0658	C_16_H_12_O_7_	317.0681	^26^
10.	Isorhamnetin 3-*O*-*β*-glucopyranoside-4՝-*O*-*β*-xylopyranoside	8.3	610.15462	611.1619	C_27_H_30_O_16_	285.0398 302.0408 317.0653 479.1168	^27^
11.	**11a**- Vitexin	8.3	432.10582	433.1131	C_21_H_20_O_10_	313.0714 323.0925 337.0699 367.0820	
	**11b**- Isovitexin						
12.	8-(1,1-Dimethylallyl) kaempferide	8.5	368.11102	369.1183	C_21_H_20_O_6_	91.0533 193.0860	^29^
13.	**13a**- Vitexin 2՝՝-*O*-rhamnoside	10.7	578.15522	579.1625	C_27_H_30_O_14_	301.0732 302.0771 319.088 342.1009	^30^
	**13b**- Isovitexin 2՝՝-*O*-rhamnoside						^31^
14.	Isorhamnetin 3-*O*-*α*-L-rhamnopyranosyl-(1→2)-*α*-L-arabinopyranoside	16.8	594.40082	595.4081	C_27_H_30_O_15_	301.2066 317.1797 335.1924 336.1930	^32^
Phenolics							
15.	Coumarin	1.4	146.03672	147.044	C_9_H_6_O_2_	65.0361 91.0541 119.0492	^33^
16.	Precocene II	3.9	220.12092	221.1282	C_13_H_16_O_3_	87.0441 91.0516 107.0499 135.0920	^34^
17.	Sinapic acid	7.1	224.13162	225.1389	C_11_H_12_O_5_	53.0380 65.0380 91.0527 92.0573 225.1385	^21,22^
18.	Myricoside	7.1	756.21482	757.2221	C_33_H_40_O_20_	283.0601 313.0709 337.0722 757.2195	^35^
19.	Icariside D2	10.7	300.06862	301.0759	C_14_H_20_O_7_	55.0542 301.0762 302.0745	^36^
20.	*p*-Coumaric acid glucoside	12.1	326.07072	327.0780	*C*_*15*_ *H*_*18*_ *O*_*8*_	-	^37^
21.	(+) Sinapoyl malate (Hydroxycinnamic acid)	16.7	340.14592	341.1532	C_15_H_16_O_9_	149.0015 323.1394	^19,21^
Sesquiterpenoids							
22.	Tutin	1.7	294.10632	295.1136	C_15_H_18_O_6_	97.0279 152.0332 197.0923	^38^
23.	**23a**- Linifolin A	2.1	304.12712	305.1344	C_17_H_20_O_5_	209.0922 227.1024 287.1230	^39^
	**23b**- Arteglasin A						^40^
24.	Orizabin	6.5	366.14312	367.1504	C_19_H_26_O_7_	144.0813 188.0712 229.0966 349.1390	^41^
25.	Mexicanin E	6.7	232.12142	233.1287	C_14_H_16_O_3_	65.0387 91.0533 92.0566 215.1236	^42^
26.	Saupirin	7.4	346.11702	347.1243	C_19_H_22_O_6_	174.0950 193.0771 311.1014 329.1154	^43^
27.	**27a**- Artabsin	7.6	248.15282	249.1601	C_15_H_20_O_3_	91.0532 135.0913 203.1548	^44,45^
	**27b**- Artemorin						^44,45^
28.	**28a**- Graminiliatrin	10.3	434.16052	435.1678	C_22_H_26_O_9_	265.0468 267.0588 277.0525 390.1096	^46^
	**28b**- Eleganin						
Nitrogenous compounds							
29.	Adenine	1.5	135.06792	136.0752	C_5_H_5_N_5_	65.0392, 91.0535, 136.0758	^36^
30.	Asparagine	1.7	132.05302	133.0603	C_4_H_8_N_2_O_3_	70.0290 74.0239 87.0547	^47^
31.	Adenosine	1.7	267.09562	268.1029	C_10_H_13_N_5_O_4_	60.0446 130.0493 172.0606 184.0611	^21,36^
32.	*N*-Methylnicotinate (Trigonelline)	1.8	137.04702	138.0543	C_7_H_7_NO_2_	50.0155 53.0386 94.0649 110.0588	^48^
33.	Picolinic acid	2.9	123.03132	124.0386	C_6_H_5_NO_2_	51.0230 52.0179 53.0387 80.0497	^49^
34.	Sinapine (Sinapoylcholine)	4.2	309.12112	310.1284	C_16_H_24_NO_5_^+^	264.1241	^21,22^
35.	**35a**- Caulilexin C (1-Methoxyindole-3-acetonitrile)	4.6	186.02942	187.0367	C_11_H_10_N_2_O	112.9986	^36^
	**35b**- Arvelexin (4-Methoxyindole-3-acetonitrile)						^36^
36.	Methyl-1-methoxy-1H-indole-3-acetate	6.5	219.08992	220.0972	C_12_H_13_NO_3_	65.0381 91.0526 91.0557 220.0992	^36^
Essential oil compounds							
37.	**37a-** gamma-Thujaplicin	1.4	164.08372	165.091	C_10_H_12_O_2_	92.0576 93.0626 119.0851 120.0892	^50^
	**37b-** Eugenol						^51^
38.	Myristicin	4.3	192.09012	193.0974	C_11_H_12_O_3_	65.0392 91.0530 147.0923	^52^
39.	Acetyleugenol	8.2	206.09812	207.1054	C_12_H_14_O_3_	55.0545 111.0788 147.0850 175.0781	^53^
Isothiocyanates							
40.	3-(Methylsulphinyl) propyl isothiocyanate (Iberin)	1.5	163.10302	164.1103	C_5_H_9_NOS_2_	55.0545 81.0709 100.1106	^54^
41.	4-(Methylthio) butanenitrile (Iberverin nitrile)	1.8	115.06362	116.0709	C_5_H_9_NS	68.0496 116.0698	^54^
Fatty acids							
42.	Macaene (5-Oxo-6*E*,8*E*-octadecadienoic acid)	1.6	294.15772	295.165	C_18_H_30_O_3_	116.928 152.9949 185.1185 279.2329	^55^
43.	*α*-Licanic acid	9.8	292.20382	293.2111	C_18_H_28_O_3_	55.0537 67.0536 275.2029 276.2018	^56^
Lignans							
44.	(-)-Nyasol (cis-Hinokiresinol)	4.2	252.11122	253.1185	C_17_H_16_O_2_	79.0545 107.0482 130.0493 147.0759	^57^
45.	Randainol	8.2	282.13732	283.1446	C_18_H_18_O_3_	79.0538 91.0534 103.0532 131.0496	^58^
Triterpene							
46.	Papyriferic acid	7.5	604.35142	605.3587	C_35_H_56_O_8_	111.0432 129.0561 155.0743 588.3327	^59^
Phytolactone							
47.	Dihydromethysticin	1.7	276.09592	277.1032	C_15_H_16_O_5_	130.0499 147.0760 150.0834 214.1123	^60^
Alkane							
48.	Eicosane (Icosane)	8.2	282.13732	283.1446	C_20_H_42_	103.0532 237.1401 283.1435	^21^
Iridoid glycoside							
49.	7-Dehydrologanin tetraacetate	10.7	556.17272	557.1800	C_25_H_32_O_14_	201.0469 219.0551 279.0937 280.0971	^61^

**Table 4:** Antioxidant activity of *M. grandiflora* Kuntze extracts using DPPH, ABTS and FRAP assays.

*M. grandiflora* Extract/Standard	DPPH IC_50_ (µg/mL)	ABTS IC_50_ (µg/mL)	FRAP (mmol AA/g)†
*n*-Hexane extract	1120^***, ###^	36.41 ± 1.07^***, ###^	1.41 ± 0.05
Defatted extract	835.23^***^	25.67 ± 0.75^***^	1.76 ± 0.06
Ascorbic acid	68.67	12.13 ± 0.35	

Data represents SEM where ***P < 0.001 considered statistically significant compared to ascorbic acid treatment while ###*P* < 0.001 considered statistically significant compared to defatted extract treated group using analysis of variance (ANOVA) followed by Tukey’s as post ANOVA test using version eight of GraphPad Prism (La Jolla, CA, USA).

†mmol ascorbic acid equivalent in 1 g of dry extract.

**Table 5:** Anti-inflammatory and cytotoxic activities of *M. grandiflora* Kuntze extracts.

Extract/Standard	Anti-inflammatory activity (IC_50_ µg/mL)			Cytotoxic activity (IC_50_ µg/mL)	
	COX-1	COX-2	SI†	Caco-2	MCF-7
*n*-Hexane extract	5.208± 0.22^###,@@@^	3.391± 0.15^###,$$$,@@@^	1.54	2.892 ± 0.110^###^	3.855 ± 0.120^***,###^
Defatted extract	8.658± 0.28^$$$,@@@^	1.723± 0.06^$$$,@@@^	5.02	15.070 ± 0.820^***^	7.271 ± 0.420^***^
Staurosporine^®^	-	-	-	2.016 ± 0.098	13.630 ± 0.660
Ibuprofen^®^	6.195± 0.23^@@@^	2.398± 0.09^@@@^	2.58	-	-
Celecoxib^®^	21.500 ± 0.69	0.786 ± 0.030	27.40	-	-

Data denote mean ± SEM where ***P < 0.001 considered statistically significant compared to Staurosporine treated group, ^###^*P* < 0.001 considered statistically significant compared to defatted extract treated group, ^$$$^*P* < 0.001 considered statistically significant compared to ibuprofen treated group while ^@@@^*P* < 0.001 considered statistically significant compared to celecoxib treated group using analysis of variance (ANOVA) followed by Tukey’s as post ANOVA test using version eight of GraphPad Prism (La Jolla, CA, USA).

*MCF-7*: Breast carcinoma cell lines, *Caco-2*: Colon carcinoma cell lines.

*Selectivity index (SI) = IC_50_ (COX-1)/IC_50_ (COX-2).

**Table 6:** Docking scores (kcal/mol) of the annotated compounds from the *n*-hexane extract by GC-MS and the defatted extract by LC-HRMS analyses of *M. grandiflora* Kuntze against CDK enzymes.

No.	Compound	Binding score (kcal/mol)		
		CDK2	CDk4	CDK6
Compounds annotated from GC-MS analysis of the *n*-hexane extract				
**1**	1,7-Octadiene, 2,5-bis-(*cis*) -(2,2-dimethyl-3-carboxycyclopropyl)	−8	−7.1	−7.1
**2**	Hexadecanoic acid, methyl ester (Palmitic acid, methyl ester)	−6.8	−5.3	−5.3
**3**	9-Tetradecynoic acid, methyl ester or (+)-(*Z*)-Longipinane)	−7.1	−6	−6.7
**4**	9,12,15-Octadecatrienoic acid, methyl ester (Linolenic acid, methyl ester)	−7.5	−6.3	−6.2
**5**	2-Hexadecen-1-ol, 3,7,11,15-tetramethyl-, acetate, [R-[R*,R*-(*E*)]	−7.6	−6.6	−6.5
**6**	13-Docosenamide, (*Z*)	−7.1	−5.9	−6.7
**9**	Ethyl 9, 12, 15 -octadecatrienoate	−7.3	−6.1	−5.8
**10**	Hexadecanedioic acid	−6.8	−5.8	−5.4
**12**	Tricyclo[5.3.1.0(6,11)]undecane-11-carboxylic acid, 1,5,5-trimethyl-8-oxo	−5.6	−7.2	−7.5
**13**	Methyl 3-*cis*,9-*cis*,12-*cis*-octadecatrienoate	−7.1	−6.4	−5.9
**14**	Tetraacetyl-*d*-xylonic nitrile	−5.5	−6.1	−5.7
**15**	*Cis*-vaccenic acid (*cis*−11-Octadecenoic acid)	−6.8	−5.5	−5.6
**16**	Benzene, (1-hexyloctyl)-	−7.7	−6.4	−6.4
**18**	Octahydrobenzo[*β*]pyran,4*α*-acetoxy-5,5,8*α*,-trimethyl	−4.8	−5.4	−5.2
**19**	5-Benzofuranacetic acid,6-ethenyl − 2,4,5,6,7,7*α*-hexahydro-3,6-dime	−6.7	−6.8	−8
**20**	Phen-1,4-diol, 2,3-dimethyl-5-trifluoromethyl	−7.5	−7.2	−6.5
Compounds annotated from UPLC-HRMS analysis of the defatted extract				
**1**	**1a-** Isobavachalcone	−9.8	−9.3	−8.6
	**1b-** Glabranin (8-prenylpinocembrin)	−9.4	−8.6	−9.1
**2**	Apigeninidin	−9.7	−8.8	−8.5
**3**	Quercetin-3-sophoroside-7-glucoside	−7.6	−9.1	−8.7
**4**	**4a**- Vitexin 2՝՝-*O*-*β*-D-glucoside	−7.7	−8.7	−9.6
	**4b**- Isovitexin 2՝՝-*O*-*β*-D-glucoside (saponarin)	−9.4	−9	−8.6
**5**	Quercetin 3-*O*-*β*-D-glucopyranosyl-7-*O*-*α*-L-rhamnopyranoside	−7.8	−9.5	−9.1
**6**	**6a-**Isorhamentin-3-*O-β*-glucopyranoside-7-*O-α*-rhamnopyranoside	−9.1	−9.2	−8.2
	**6b-** Isorhamnetin 3-*O*-rutinoside (Narcissin)	−8.8	−8.8	−8.4
**7**	**7a-** Luteolin 8-*C-β*-glucoside (Orientin)	−8.4	−8.3	−9.3
	**7b-** Luteolin 6-*C-β*-glucoside (Isoorientin)	−10.3	−9.3	−8.9
**8**	Isoscoparin (Chrysoeriol 6-*C*-glucoside)	−9	−9	−7.7
**9**	Isorhamnetin	−9.4	−8.6	−7.9
**10**	Isorhamnetin 3-*O-β*-glucopyranoside-4՝-*O-β*-xylopyranoside	−7.6	−8.5	−7.4
**11**	**11a**- Vitexin	−8.2	−8.3	−9.2
	**11b**- Isovitexin	−9.2	−9.4	−8.5
**12**	8-(1,1-Dimethylallyl) kaempferide	−8.4	−9.2	−8.7
**13**	**13a**- Vitexin 2՝՝-*O*-rhamnoside	−7.8	−9.4	−9.2
	**13b**- Isovitexin 2՝՝-*O*-rhamnoside	−9.5	−9.8	−8.6
**14**	Isorhamnetin 3-*O-α*-L-rhamnopyranosyl-(1→2)-*α*-L-arabinopyranoside	−8.1	−8.3	−7.9
**15**	Coumarin	−7.6	−6.7	−6.7
**16**	Precocene II	−7.3	−6.7	−6.5
**17**	Sinapic acid	−7	−6	−6.1
**18**	Myricoside	−8.7	−9.6	−8.2
**19**	Icariside D2	−7.7	−6.9	−7.4
**20**	*p*-Coumaric acid glucoside	−8.2	−7.2	−7.9
**21**	(+) Sinapoyl malate (Hydroxycinnamic acid)	−7.8	−6.9	−7.9
**22**	Tutin	−5.8	−7	−6.4
**23**	**23a**- Linifolin A	−5.7	−8.9	−7
	**23b**- Arteglasin A	−8	−8.5	−7.7
**24**	Orizabin	−6.8	−9.2	−7.8
**25**	Mexicanin E	−7.8	−8.4	−8.1
**26**	Saupirin	−7.9	−8.5	−8.6
**27**	**27a**- Artabsin	−7.5	−8.4	−8.4
	**27b**- Artemorin	−7.3	−8.6	−8.1
**28**	**28a**- Graminiliatrin	−7.9	−8.7	−9.2
	**28b**- Eleganin	−7.2	−8.6	−7.7
**29**	Adenine	−5.7	−5.2	−4.7
**30**	Asparagine	−4.8	−4.9	−4.3
**31**	Adenosine	−6.4	−6.8	−6.8
**32**	*N*-Methylnicotinate (Trigonelline)	−5.7	−5.3	−5.4
**33**	Picolinic acid	−5.6	−5.4	−4.9
**34**	Sinapine (sinapoylcholine)	−7.4	−6.5	−6.3
**35**	**35a**- Caulilexin C (1-Methoxyindole-3-acetonitrile)	−7.7	−6.6	−6.8
	**35b**- Arvelexin (4-Methoxyindole-3-acetonitrile)	−7.1	−6.8	−6.5
**36**	Methyl-1-methoxy-1H-indole-3-acetate	−7.5	−6.7	−6.8
**37**	**37a-** gamma-Thujaplicin	−7.7	−6.4	−6.6
	**37b-** Eugenol	−6.8	−6.7	−5.8
**38**	Myristicin	−7.5	−6.5	−6.1
**39**	Acetyleugenol	−7.5	−6.7	−6.2
**40**	3-(Methylsulphinyl) propyl isothiocyanate (Iberin)	−4.1	−3.6	−3.6
**41**	4-(Methylthio) butanenitrile (Iberverin nitrile)	−4.1	−3.6	−4
**42**	Macaene (5-Oxo-6*E*,8*E*-octadecadienoic acid)	−7.2	−6	−7.2
**43**	*α*-Licanic acid	−7.4	−6.6	−6.1
**44**	(-)-Nyasol (*cis*-Hinokiresinol)	−9.4	−7.8	−7.8
**45**	Randainol	−9.7	−8.3	−7.9
**46**	Papyriferic acid	−7.1	−8.7	−7.4
**47**	Dihydromethysticin	−9.3	−8.3	−7.7
**48**	Eicosan (Icosane)	−6.9	−5.6	−6
**49**	7-Dehydrologanin tetraacetate	−7	−7.9	−7.6
	**WN333** ^**a**^	−10.2	−8	−7.6
	**Atirmociclib** ^**b**^	−7.6	−9.8	−9.2

^a^Co-crystallized ligand of CDK2.

^b^Co-crystallized ligand of CDK4 and CDK6.

## Supplementary Information

Below is the link to the electronic supplementary material.

Supplementary Material 1

## Data Availability

All data generated or analyzed during this study are included in this published article and supplementary data.
