# Supplementary material for: Multi-analytical and biological insights into Malcolmia grandiflora Kuntze
Source: Sci Rep. 2026 Apr 21;16:13022. doi: 10.1038/s41598-026-44715-x (PMC13100116; doi:10.1038/s41598-026-44715-x)
Supplement: Supplementary file 1 — Supplementary Material 1 [file 41598_2026_44715_MOESM1_ESM.docx]

**Multi-Analytical and Biological Insights into Malcolmia grandiflora Kuntze**

Ahlam Hashem Elwekeel^1^, Enas I. A. Mohamed^1^, Elham Amin^2*^, Malak Sadek^3^, Mahytab Mohamed, and Marwa H. A. Hassan^1*^

^1^Department of Pharmacognosy, Faculty of Pharmacy, Beni-Suef University, Beni-Suef 62514, Egypt.

^2^Department of Pharmaceutical Chemistry and Pharmacognosy, College of Pharmacy, Qassim University, Buraidah 52571, Saudi Arabia.

^3^Faculty of Medicine, Beni-Suef University, Beni-Suef 62514, Egypt.

**Ahlam** **Hashem Elwekeel:** [ahlam_hasham2000@yahoo.com](mailto:ahlam_hasham2000@yahoo.com), [0000-0001-7954-6310](https://www.scopus.com/redirect.uri?url=https://orcid.org/0000-0001-7954-6310&authorId=54941030500&origin=AuthorProfile&orcId=0000-0001-7954-6310&category=orcidLink)

**Enas I. A. Mohamed**: [enas.mohamed@pharm.bsu.edu.eg](mailto:enas.mohamed@pharm.bsu.edu.eg), [0000-0002-6943-9383](https://www.scopus.com/redirect.uri?url=https://orcid.org/0000-0002-6943-9383&authorId=57194003150&origin=AuthorProfile&orcId=0000-0002-6943-9383&category=orcidLink)

**Elham Amin:** [el.saleh@qu.edu.sa](mailto:el.saleh@qu.edu.sa), 0000-0002-7190-3974

**Malak Sadek**: [Malaksadek.203@gmail.com](mailto:Malaksadek.203@gmail.com), 0009-0008-8375-2481

**Mahytab Mohamed**: [mahitabmuhammadsadiq547_sd23@med.bsu.edu.eg](mailto:mahitabmuhammadsadiq547_sd23@med.bsu.edu.eg), 0009-0006-4434-6072

**Marwa H. A. Hassan**: [mh_elsefy@yahoo.com](mailto:mh_elsefy@yahoo.com), [marwa.hassan@pharm.bsu.edu.eg](mailto:marwa.hassan@pharm.bsu.edu.eg), 0000-0002-0952-5984

^*^**Correspondence:**

Marwa H. A. Hassan, [marwa.hassan@pharm.bsu.edu.eg](mailto:marwa.hassan@pharm.bsu.edu.eg), 0000-0002-0952-5984

Elham Amin, el.saleh@qu.edu.sa, 0000-0002-7190-3974

Mobile No.: +201278982288

**Supplementary material:**

**Experimental section:**

**Chemicals**

1,1-Diphenyl-2-picrylhydrazyl (DPPH), 2,4,6-tri(2-pyridyl)-s-triazine (TPTZ), ABTS**^·+^** [(2,2'-Azino-bis (3-ethylben-zothiazoline-6-sulfonic acid) diammonium salt], ascorbic acid, Folin–Ciocalteu reagent, gallic acid, and quercetin were purchased from Sigma Chemical Co. (St. Louis, MO, USA).

**Total Phenolic content (TPC)**

Folin–Ciocalteu method was used for determination of the total phenolic content (TPC) of the alcoholic extract by the method [^1^](#_ENREF_1). A 0.1 ml aliquot of diluted extract solution (1 mg/mL) and 0.75 mL of Folin–Ciocalteu reagent (10% in distilled water) were mixed, after 5 minutes 0.75 mL of 6% sodium carbonate was mixed. The mixture was shaken and kept for 90 min in the dark at room temperature. Blue color was developed which measured by spectrophotometer at 725 nm. Calibration curve of gallic acid was plotted and TPC was expressed as mg gallic acid equivalent/gram dry extract.

**Total flavonoid content (TFC)**

Aluminum chloride was used for determination of TFC content in the alcoholic extract according to the method reported by [^2^](#_ENREF_2). 0.5 mL of the extract (1 mg/mL) was mixed with 0.5 mL of 2 % aluminum chloride; the mixture was kept for 1hr at room temperature. Absorbance of the developed color was measured at 420 nm by a spectrophotometer. The calibration curve of quercetin was plotted, TFC was expressed as mg of quercetin /gram dry extract.

**GC-MS analysis**

GC/MS analysis of the *n*-hexane extract using a Shimadzu GC/MS-QP 2010 chromatograph (Kyoto, Japan) which supplied with a capillary column DB-5 (30 m × 0.25 mm i.d. × 0.25 μm film thickness; Restek, Bellefonte, PA, USA). The oven temperature was adjusted at 50 °C for 3 min, then raised to 300 °C with rate 5 °C/min, held constant at 300 °C for 10 min; the injector temperature was set at 280 °C. The carrier gas used was helium with a flow rate of 1.37 mL/min. Diluted sample (1% v/v) was injected at a split ratio of 15:1 in a volume of 2 μL. The ion source temperature was set at 220 °C and the interface temperature at 280 °C. The electron impact ionization (EI) was 70 eV and the mass spectra were analyzed in the scan mode over the range of 35 to 500 amu. Identification of the chemical constituents was performed by cross-referencing their retention times and mass fragmentation patterns with the Wiley and National Institute of Standards and Technology (NIST) mass spectral library databases.

**LC-HRMS analysis**

The separation of compounds occurred in the defatted extract was achieved using a Thermo Scientific C18 column (Acclaim TM Polar Advantage II, 3 x 150 mm, 3µm particle size) connected to an UltiMate 3000 UHPLC system (Dionex). A gradient elution at a flow rate of 0.4 mL/min and the column temperature of 40 °C was employed. The mobile phase consisted of water containing 0.1% formic acid (A) and 100% acetonitrile (B), with a total run time of 22 mins. The sample injection volume was 3 µL. The gradient program started at 5% B, increased to 80% B over 15 mins, and then returned to 5% B for the final 7 mins (15-22 min).

High-resolution mass spectrometry analysis was performed using a MicroTOF QIII Bruker Daltonic instrument with positive electrospray ionization (ESI). The ESI source parameters were set as follows: capillary voltage at 4500 V, nebulizer pressure at 2.0 bar, and drying gas with flow rate of 8 L/min at a temperature of 300 °C. Mass spectra of interest were acquired over a mass-to-charge (*m/z*) range of 50-1000. Molecular ion peaks were obtained from the TOF analyzer, and processed using Compass Data Analysis software (Bruker Daltonik GmbH) [^3^](#_ENREF_3)^,^[^4^](#_ENREF_4).

***In vitro* biological assays**

**Antioxidant assays**

**DPPH method:**

DPPH was used for assessment of antioxidant activity of the hexane and defatted extracts according to[^5^](#_ENREF_5). 100 µL of the different concentration of the extract and ascorbic acid (25-400 µg/mL) was mixed with 1.9 mL of DPPH solution (3.4 mg/100 mL ethanol), the mixtures were incubated for 30 min in the dark at room temperature. The developed color was measured at 517 nm by spectrophotometer; the result was expressed as IC_50_ for the extracts and ascorbic acid.

**ABTS method:**

The stock solution of ABTS was prepared by mixing 7.4 mM ABTS solution and 2.6 mM potassium persulfate solution in the ratio of 1:1and allowed to react for 12 h at room temperature in the dark. 3 mL of the stock solution was diluted with methanol to 100 mL. Different concentrations of the extracts and ascorbic acid was prepared (8-250 µg/mL). 150 µl of the different concentration sample/ standard (ascorbic acid) was mixed with 2.85 ml of the diluted ABTS reagent, the mixture kept in the dark for 30 min then the absorbance was recorded at 734 nm. The results were reported as IC_50_ of the samples and ascorbic acid[^6^](#_ENREF_6).

**FRAP method:**

Fresh FRAP reagent was prepared by mixing 300 mM acetate buffer,10 mM TPTZ (2,4,6-tripyridyl-S-triazine) solution in

40 mM HCl, and 20 mM FeCl3.6H2O (10:1:1:1), 150 µl of the sample or the standard (ascorbic acid) was mixed with 2.85 ml of the reagent, incubate at 37°C for 30 minutes in the dark, the absorbance was measured at 593 nm against a reagent blank (FRAP reagent + solvent), the results calculated as mmol ascorbic acid/ gram of the extract[^7^](#_ENREF_7).

**Anti-inflammatory Activity**

The ability of the *n*-hexane and defatted extracts to inhibit COX-1 and COX-2 enzymes was tested and compared with known inhibitors such as celecoxib and indomethacin, which served as positive controls. This assay used commercially available enzyme immunoassay kits designed for human COX-1 and COX-2 inhibition screening, and the procedures followed the manufacturer's instructions (catalog numbers 701070 and 701080 from Cayman Chemical).

For the experiment, the test samples were dissolved in DMSO. Various concentrations of these samples, along with the COX-1, COX-2 enzymes and heme, were incubated separately for 10 minutes at 37 °C in a specific reaction buffer. The enzymatic reaction was triggered by adding 100 mM arachidonic acid and immediately stopped after 30 seconds by adding stannous chloride. The amount of PGF2α (Prostaglandin F2α) produced was then measured using an enzyme immunoassay. The optical density of the samples was read after one hour at 450 nm using a DYNATech MR 5000 microplate reader. The percentage of the enzyme inhibition was calculated for each tested concentration and compared to a control. The IC_50_ values for both COX-1 and COX-2 were calculated from the resulting dose-response curves [^8^](#_ENREF_8)^,^[^9^](#_ENREF_9). Selectivity index (SI) was calculated. It is defined as the ratio of the IC_50_ value for the constitutive COX-1 to the IC_50_ value for the inducible COX-2. A Selectivity index value greater than 1 indicates a higher specificity for the COX-2 enzyme, suggesting a potentially lower risk of gastrointestinal side effects associated with COX-1 inhibition.

**Cytotoxic activity**

The cytotoxic activity of the *n*-hexane and defatted extracts were examined using a cell viability assay, following a method previously described by Mosmann in 1983 [^10^](#_ENREF_10)^,^[^11^](#_ENREF_11). Two cancer cell lines MCF-7 (breast carcinoma) and Caco-2 (colon adenocarcinoma) obtained from the American Type Culture Collection (ATCC) that was supplied by Vacsera, Egypt, were used for the experiment. A Serial dilution (100, 25, 6.3, 1.6 and 0.4 µg/mL) of both extracts were prepared and solved in DMSO, then estimated for their abilities to inhibit the growth of cancer cells.

In brief, the cancer cell lines were suspended in their respective growth media and seeded at a concentration of 5 x 10^4^ cells per well in 96-well plates and incubated for 24-hrs. Different concentrations of the *n*-hexane and defatted extracts of *M. grandiflora* were added to the wells (in triplicate). For each plate, six control wells containing only media or media with 0.5% DMSO (the solvent for the extract) were included. After a further 24-hrs of incubation, the number of viable cells was counted using the MTT assay.

For the MTT assay, the media was removed and replaced with fresh RPMI 1640 medium (without phenol red) and an MTT stock solution. The plates were incubated at 37 °C with 5% CO_2_ for 4 hrs. Then, 85 µl of the media was removed, and 50 µl of DMSO was added to each well to solubilize the formazan crystals formed by viable cells. After a 10-minute incubation at 37 °C, the optical density (OD) was measured at 590 nm using a microplate reader (SunRise, TECAN, Inc, USA).

The percentage of cell viability was calculated using the formula:

[OD of treated cells / OD of control cells] x 100%.

Survival curves were plotted by graphing the relationship between surviving cells and the extract concentration for each cancer cell line. The IC_50_ value, defined as the concentration of the extract that was required to inhibit 50% of cell growth, was estimated from these dose-response curves using Graphpad Prism software (San Diego, CA. USA). Staurosporine, a known anticancer drug, was used as a positive control. The reagents used for cell culture included Fetal Bovine serum, DMEM, RPMI-1640, HEPES buffer, L-glutamine, gentamycin, and Trypsin-EDTA, which were purchased from Lonza.

**References**

1 Velioglu, Y., Mazza, G., Gao, L. & Oomah, B. Antioxidant activity and total phenolics in selected fruits, vegetables, and grain products. *Journal of agricultural and food chemistry* **46**, 4113-4117 (1998).

2 Mbinda, W. & Musangi, C. Antioxidant activity, total phenolic and total flavonoid. *J Phytopharmacol* **8**, 161-166 (2019).

3 Abdulhafiz, F. *et al.* LC–TOF-MS/MS and GC-MS based phytochemical profiling and evaluation of wound healing activity of *Oroxylum Indicum* (L.) Kurz (Beka). *Frontiers in Pharmacology* **13**, 1050453 (2022).

4 Amin, E., Abdel-Bakky, M. S., Mohammed, H. A. & Hassan, M. H. Chemical profiling and molecular docking study of *Agathophora alopecuroides*. *Life* **12**, 1852 (2022).

5 Kulisic, T., Radonic, A., Katalinic, V. & Milos, M. Use of different methods for testing antioxidative activity of oregano essential oil. *Food chemistry* **85**, 633-640 (2004).

6 Iqbal, E., Salim, K. A. & Lim, L. B. Phytochemical screening, total phenolics and antioxidant activities of bark and leaf extracts of *Goniothalamus velutinus* (Airy Shaw) from Brunei Darussalam. *Journal of King Saud University-Science* **27**, 224-232 (2015).

7 Thaipong, K., Boonprakob, U., Crosby, K., Cisneros-Zevallos, L. & Byrne, D. H. Comparison of ABTS, DPPH, FRAP, and ORAC assays for estimating antioxidant activity from guava fruit extracts. *Journal of food composition and analysis* **19**, 669-675 (2006).

8 Ahmed, E. M., Kassab, A. E., El-Malah, A. A. & Hassan, M. S. Synthesis and biological evaluation of pyridazinone derivatives as selective COX-2 inhibitors and potential anti-inflammatory agents. *European Journal of Medicinal Chemistry* **171**, 25-37 (2019).

9 Amin, E., Elwekeel, A., Alshariedh, N. F., Abdel-Bakky, M. S. & Hassan, M. H. GC-MS Analysis and bioactivities of the essential oil of *Suaeda aegyptiaca*. *Separations* **9**, 439 (2022).

10 Mosmann, T. Rapid colorimetric assay for cellular growth and survival: application to proliferation and cytotoxicity assays. *Journal of immunological methods* **65**, 55-63 (1983).

11 Gomha, S. M., Riyadh, S. M., Mahmmoud, E. A. & Elaasser, M. M. Synthesis and anticancer activities of thiazoles, 1, 3-thiazines, and thiazolidine using chitosan-grafted-poly (vinylpyridine) as basic catalyst. *Heterocycles* **91**, 1227-1243 (2015).
